# Antioxidant-Based Medicinal Properties of Stingless Bee Products: Recent Progress and Future Directions

**DOI:** 10.3390/biom10060923

**Published:** 2020-06-18

**Authors:** Mohammad A. I. Al-Hatamleh, Jennifer C. Boer, Kirsty L. Wilson, Magdalena Plebanski, Rohimah Mohamud, Mohd Zulkifli Mustafa

**Affiliations:** 1 Department of Immunology, School of Medical Sciences, Universiti Sains Malaysia, Kubang Kerian 16150, Kelantan, Malaysia; alhatamleh@student.usm.my (M.A.I.A.-H.); rohimahm@usm.my (R.M.); 2 Translational Immunology and Nanotechnology Unit, School of Health and Biomedical Sciences, RMIT University, Bundoora 3083, Australia; jennifer.boer@rmit.edu.au (J.C.B.); kirsty.wilson2@rmit.edu.au (K.L.W.); magdalena.plebanski@rmit.edu.au (M.P.); 3 Hospital Universiti Sains Malaysia, Kubang Kerian 16150, Kelantan, Malaysia; 4 Department of Neurosciences, School of Medical Sciences, Universiti Sains Malaysia, Kubang Kerian 16150, Kelantan, Malaysia

**Keywords:** stingless bee, meliponines, honey, propolis, natural products, phenolic compounds, flavonoids, antioxidants

## Abstract

Stingless bees are a type of honey producers that commonly live in tropical countries. Their use for honey is being abandoned due to its limited production. However, the recent improvements in stingless bee honey production, particularly in South East Asia, have brought stingless bee products back into the picture. Although there are many stingless bee species that produce a wide spread of products, known since old eras in traditional medicine, the modern medical community is still missing more investigational studies on stingless bee products. Whereas comprehensive studies in the current era attest to the biological and medicinal properties of honeybee (*Apis mellifera*) products, the properties of stingless bee products are less known. This review highlights for the first time the medicinal benefits of stingless bee products (honey, propolis, pollen and cerumen), recent investigations and promising future directions. This review emphasizes the potential antioxidant properties of these products that in turn play a vital role in preventing and treating diseases associated with oxidative stress, microbial infections and inflammatory disorders. Summarizing all these data and insights in one manuscript may increase the commercial value of stingless bee products as a food ingredient. This review will also highlight the utility of stingless bee products in the context of medicinal and therapeutic properties, some of which are yet to be discovered.

## 1. Introduction

Stingless bees (*Meliponines*) belong to the genus Apidae, which is a family of social bees from the superfamily Apoidea. Stingless bees are the highest developed species of bees that have been identified in 80 million years old parts of amber [1]. Reports of ancient populations using honey both for nutritional and medicinal properties can be traced back to nearly 5500 years ago [2]. Hand collecting honey bees was an important traditional practice in many ancient populations as it was the only way to get honey and it still persists among some people in forest areas [1]. To date there are more than 500 known stingless bee species, of which approximately 40 species have good potential as honey producers [3,4]. These species are distributed in the tropical and subtropical regions as follows: approximately 391 species in the Neotropical region of South America, 60 in the Indo-Malayan region of Asia, 50 in the Paleotropical region of Africa and 10 in the Australasia region of Australia [5]. Constructing nests in hollow tree trunks or roots, soil cavities or empty animal narrow nests is typical for many stingless bee honey (SBH) producer species [1]. As pollinators, stingless bees play an important role in the forest ecosystem by strongly influencing plant community, diversity and evolution.

**Figure 1:**
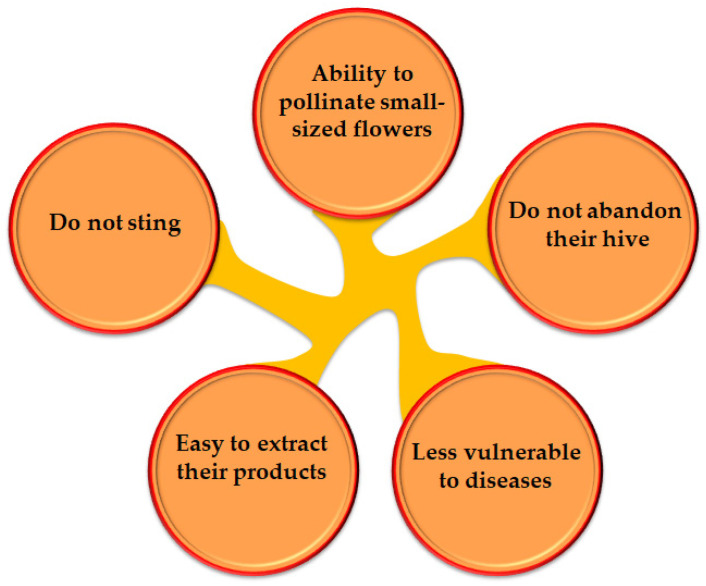
Principal characteristics of stingless bees that differentiate them from honeybees [7].

The most common species producing SBH are classified under two main genera, *Melipona* and *Trigona* [6]. While stingless bees are one of the most common types of honey producers, the distribution of SBH is lower compared to the more common honey produced by *Apis mellifera* bees (European/Western bees or honeybees) [7], which has been associated with the limited data about SBH production and its properties. Interestingly, SBH is less used as a nutritional and medicinal product [8], despite its higher nutritional and medicinal properties compared to the commonly used honeybee products (European/Western honey) [9,10,11,12]. It is believed that stingless bee products are superior promising sources of biologically active compounds over honeybees, and this can be attributed to the rich vegetation in the tropical and subtropical regions where stingless bees are found [7]. In addition, stingless bees have some principal characteristics that make them unique compared to honeybees, for example they are less vulnerable to disease (Figure 1). Recent evidence indicates that SBH has potential therapeutic benefits in several contexts, including wound healing, diabetes mellitus, eye diseases, hypertension, fertility defects, cancer, microbial infection and dysregulated lipid profiles [13]. Therefore, promoting the research on SBH would help improve the knowledge on its putative medicinal properties and ensure the conservation of SBH trade.

**Figure 2:**
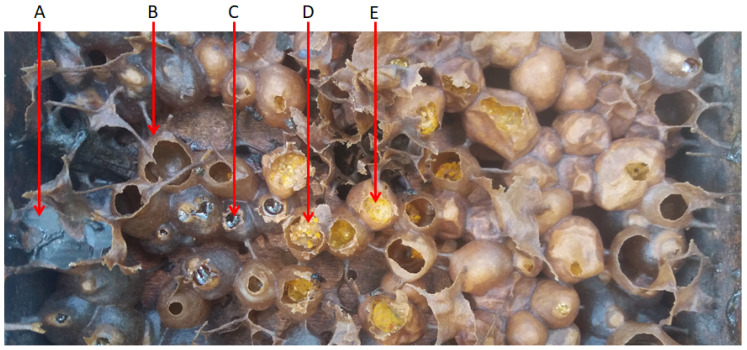
Hive box containing colony of stingless bees (*Heterotrigona itama*). A, cerumen; B, empty propolis pot; C, honey; D, bee pollen; E, fermented pollen (beebread).

**Figure 3:**
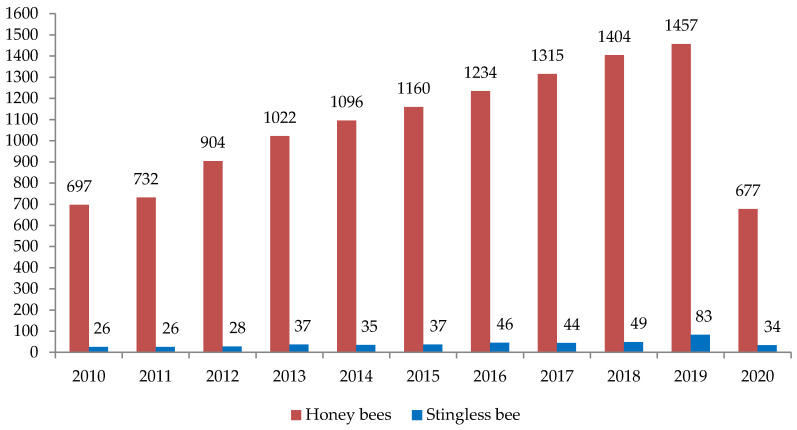
A comparison between the annual publications on both stingless bees and honey bees in the recent 10 years. Applied on PubMed database on May 9, 2020, by using search terms: (Honeybee; Honey bee; Apis; European bee; Western bee) and (Stingless bee; Melipona; Trigona).

It is important to note that stingless bee products are not restricted to honey, but can involve several products including propolis, beebread, cerumen and bee pollen (Figure 2). Although these are not as common as honey, studies have reported several medicinal properties in those products too [14,15,16]. Based on a comparison between the studies on stingless bees and honeybees, a significant gap in research on stingless bee products has been identified compared to the common honeybee products that are well-studied (Figure 3). Interestingly, in 2019, approximately double the number of studies on stingless bees were reported (Figure 3). To improve the awareness on the stingless bee, its products, properties, benefits and future opportunities, this manuscript provides a comprehensive review and recent updates on the medicinal properties of stingless bee products. We also highlight the importance of increasing research investments into stingless bee products for future medical research, as well as to motivate their production and nutritional usage.

## 2. Harvesting and Characterization of Stingless Bee Products

The percentages of stingless bee products in the beehive are still unknown; it supposed to be varied according to the species. Generally, propolis is main content of stingless bee hive, regardless the species, as the hive is constructed with it [17]. A study on nine *Trigona* species reported that the hive products of those species were 63.7% propolis, 20.9% beebread and 15.4% honey [18]. Furthermore, the proportion of use for each stingless bee product in the medicinal/commercial industry is also unknown.

Stingless bees produce their honey and store it in small resin pots in the hive. In traditional practices, honey pots were commonly squeezed to obtain the honey [19]. This method led to several disadvantages such as damaging the pot and reducing the productivity of the bees. In fact, honey obtained by squeezing the pot produced SBH contaminated with bee bread, which promoted souring of the honey and produced inconsistent honey products [20]. To overcome this, current practices in stingless beekeeping promote the hiving method with a monolayer honey pot induction system that utilizes suction pumps to aspirate the honey pot by pot without damaging the pots [21]. This method produces absolute and hygienic honey as well as supporting sustainable stingless beekeeping. However, so far there are no standard methods for harvesting other stingless bee products. Beekeepers harvest each of these products by removing it directly from the hive. Menezes et al. have suggested a good method for harvesting unfermented pollen from stingless bees by moving a colony of stingless bees to a another location and stimulate the foragers to accept a new hive, build new pots and leaving the old hive empty [22]. After a week they easily and precisely harvested the pollen from the pots of empty hive.

Despite an improvement in harvesting practices and post-harvest management of SBH, stingless bee products display variability in chemical composition associated with botanical and geographical origin, bee species and climate. Therefore, a standard characterization worldwide for stingless bee products is not available. However, Malaysia has specifically developed the first SBH standard referred to as Malaysian standard (MS) 2683:2017 [23]. It is used in local regulations according to several parameters including moisture content (<35.0%), sucrose (<7.5 g/100 g), sum of fructose and glucose (<85.0 g/100 g), maltose (<9.5 g/100 g), ash (<1.0 g/100 g), hydroxymethylfurfural (<30.0 mg/kg), pH (2.5–3.8) and identifying the plant phenolic content [23]. Additionally, this standard also set the microbial contaminant with total plate count limits at 1 × 10^3^ CFU/mL, whereas yeast, mold and coliforms must be less than 1 × 10^1^ CFU/mL [23].

## 3. Stingless Bee Honey

Given that studies on the medicinal properties of SBH are still limited with the majority being focused on common health problems (such as inflammation, cancer and microbial infection), this review outlines the studies that have utilized SBH and highlights the reported beneficial properties of SBH (Table 1). The health benefits of SBH can be broadly classified as having antioxidant, anti-inflammatory, anti-obesity, anticancer and antimicrobial properties.

### 3.1. Antioxidant Activity of SBH

**Figure 4:**
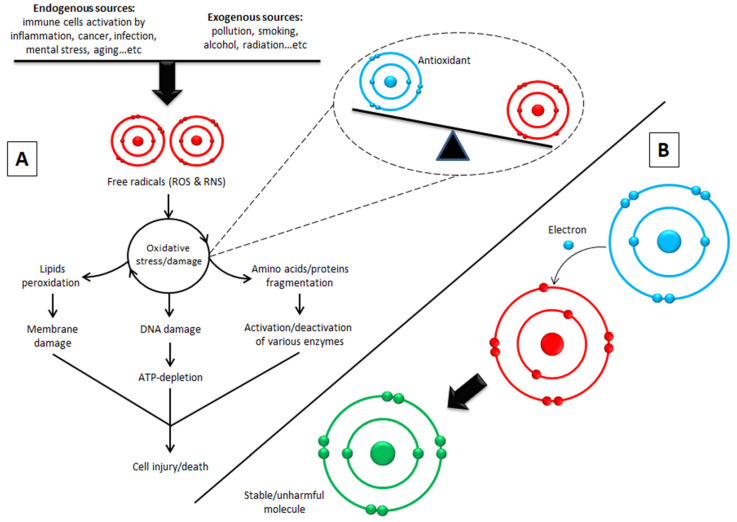
The principal of oxidative damage and the role of antioxidants in scavenging free radicals. (**A**) The free radicals generated from endogenous sources, at limited concentrations, are considered important for regulation of cell maturation, in addition to their role in immune defense. Excessive concentrations of these unstable molecules can result from illness conditions and exogenous sources, and thus lead to oxidative damage (imbalance between free radical and antioxidant concentrations). This status results in cell injury/death based on the extremely high reactivity of free radicals with vital cellular molecules including lipids, amino acids, proteins and DNA. (**B**) Antioxidants are necessary to stop the oxidative damage by neutralizing free radicals. They own this unique role due to their capability to give an electron to free radicals that have unpaired electrons to make them stable and unharmful. Phenolic compounds present in stingless bee honey products have antioxidant properties. ROS, reactive oxygen species; RNS, reactive nitrogen species [55,56].

The antioxidant activity assay for any product is based on the capability of the compounds to inhibit oxidation, thus reducing production of free radicals that result in a chain reaction causing harmful cellular alterations [50]. Studies have reported that antioxidant activity is significantly correlated with several healing properties, such as anti-inflammatory, antimicrobial, anticancer and anti-obesity activities [30,32,38,51]. This is not surprising given the role of antioxidants in oxidative stress/damage is well-known and is implicated in a wide range of diseases (Figure 4). Furthermore, the role of antioxidants is not limited to scavenging free radicals; it also have good potentials to modulate signal transduction pathways that affected by free radicals during oxidative stress and are responsible to cellular responses (e.g., inflammation, survival, proliferation and death) in various diseases [52,53]. Reinisalo et al. have shown that both the nuclear factor-erythroid-2-related factor-2 (Nrf2) pathway and intracellular cyclic AMP (cAMP) second messenger system can be modulated by antioxidant compounds [54]. Activation of Nrf2 results in activation of some Nrf2 target gene candidates (e.g., *Nrf2, SLC48A1, SLC7A11, p62, HO-1* and *Bcl-2* genes) that control antioxidant defense and autophagy. While inhibition of phosphodiesterases (PDE) promotes intracellular cAMP levels, and therefore, activation of cAMP response element-binding protein (CREB) target genes and AMP-activated protein kinase (AMPK) pathway, which is the key regulator of autophagy and also involves in regulation of Nrf2 pathway [54].

Generally, it has been suggested that the number of hydroxyl groups in the aromatic rings of antioxidants is relatively associated with their effectiveness [57]. Higher antioxidant activities have been correlated to honeys with darker colors, as the dark color derives from different pigment compounds such as flavonoids, carotenoids and phenolic compounds, the compounds that provide antioxidant properties to honey [58]. Indeed, studies from Selvaraju and others have suggested that SBH contains high concentrations of total pollen, flavonoid and phenolic contents and color intensity [30,31].

**Figure 5:**
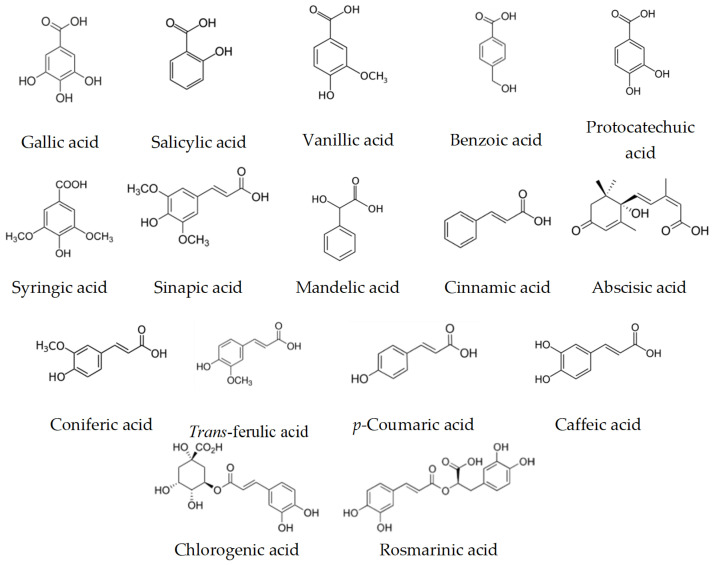
Phenolic acids have been reported in different types of SBH.

**Figure 6:**
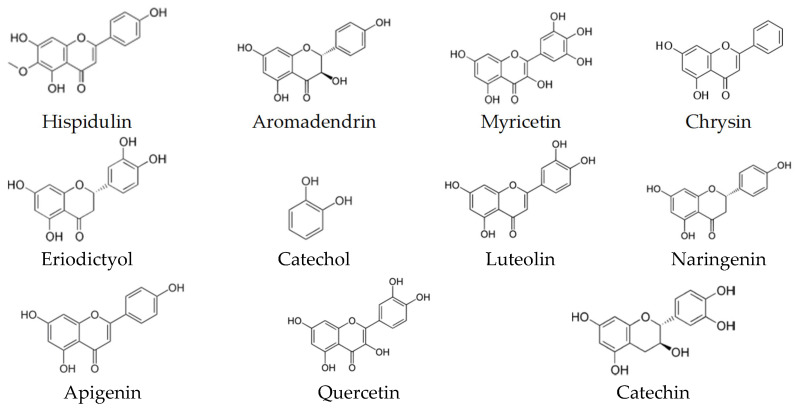

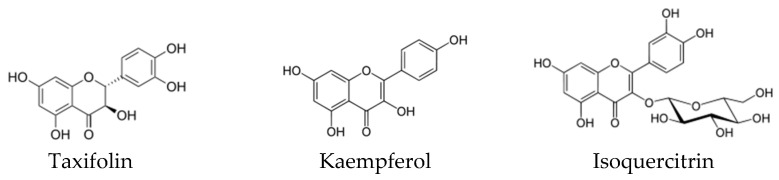
Flavonoids have been reported in different types of SBH.

**Figure 7:**
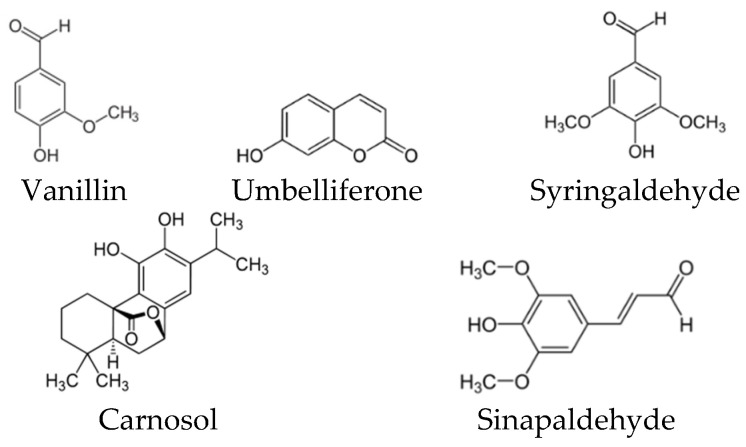
Other phenolic compounds have been reported in different types of SBH.

Many studies have explored the total phenolic content (TPC) and the total flavonoid content (TFC), calculated in mg gallic acid equivalent (GAE)/g sample, and the characterization of the phenolic compounds have been performed by different methods, especially the high performance liquid chromatography-mass spectrometric (LC-MS) [59,60]. While the most common assays to detect the antioxidant activity were DPPH (2,2-diphenyl-1-picryl-hydrazyl-hydrate)-free radical scavenging activity, ABTS (2,2′-azino-bis(3-ethylbenzothiazoline-6-sulfonic acid)) radical cation scavenging activity and ferric reducing antioxidant power (FRAP) [16,28,31,59,60]. These three assays considered as relatively inexpensiveness spectrophotometric-based tests based on color quenching or gaining of synthetic organic radicals. The colors of DPPH (purple) and ABTS (blue–green) are reducible upon exposure to antioxidants, and this decrease is negatively correlated with the measured wavelength, which, thus, refers to the antioxidant capacity [61]. While the FARP assay works in reverse; the increase in color density (decrease in wavelength) refers to higher antioxidant capacity. In this assay, ferric-tripyridyl triazine (Fe^3+^ TPTZ) is colorless and it is convertible to be ferrous (Fe^2+^) with blue color upon electron donation from antioxidants [62]. Among the several classes of phenolic compounds, a total of 16 phenolic acids (Figure 5), 19 flavonoids (Figure 6) and other five phenolic compounds (Figure 7) have been reported in SBHs at different concentrations [31,59,60,63]. The major compounds that commonly been reported are gallic acid, salicylic acid, *p*-coumaric acid, kaempferol, naringin, luteolin, catechin, apigenin and taxifolin [31,59,60,63]. The concentration of phenolic compounds differed based on the species of stingless bees and the vegetation at the sites of SBH collection [60]. These findings were supported by a study has compiled compositional data from 152 stingless bee honeys during 42 years (2006–1964) [21].

The antioxidant effects of SBH have been shown in a study conducted on a diabetic rat model. Animals underwent treatment with SBH, which caused increased levels of superoxide dismutase and glutathione (antioxidant enzymes), whereas protein carbonyl and malondialdehyde (biomarkers of oxidative stress) levels decreased in their sperms and testis, thus, improving the quality of their sperm [39]. Another study reported that the significant increase in cell viability of lymphoblastoid cell line (LCL) might be modulated by the antioxidant properties of SBH [28]. LCL viability was significantly higher during 24 h of treatment with SBH, while a decline in viability was reported after that with higher concentrations of SBH. These findings are in line with results of a comparable wound healing study on human fibroblast cells using freeze-dried SBH [36]. All together, these studies indicate that in a concentration-dependent manner, different antioxidant compounds of SBH could play a vital role in the increment or decline of cell viability by modulating oxidative stress and cytotoxicity.

### 3.2. Anti-Inflammatory Activity of SBH

**Figure 8:**
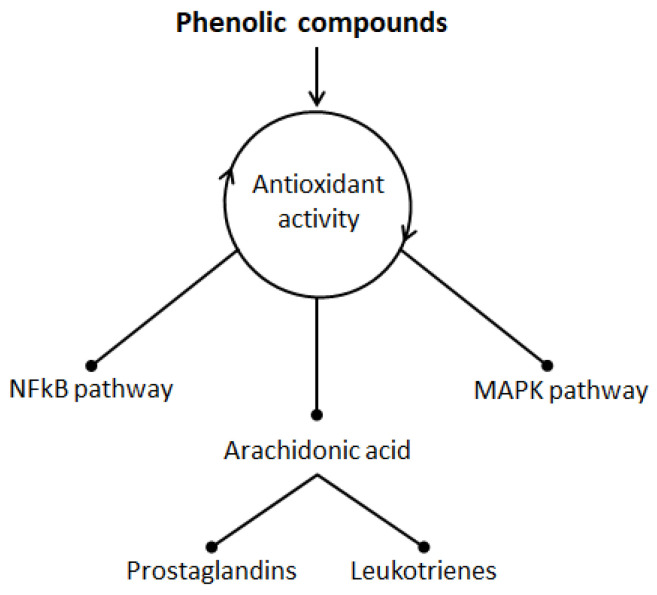
Potential mechanisms of actions for antioxidant phenolic compounds as immunomodulatory and anti-inflammatory agents [66]. These mechanisms work mainly through inhibition of two key signaling pathways; the mitogen-activated protein kinases (MAPK) and the nuclear factor kappa-light-chain-enhancer of activated B cells (NF-κB) and arachidonic acid signaling pathways. This inhibition results in complicated cellular mechanisms finished with suppression of pro-inflammatory gens, and thus block the expression of pro-inflammatory cytokines (e.g., tumor necrosis factor-alpha (TNF) and interleukin (IL)-6). In addition, the phenolic compounds also reduce the release of arachidonic acid which results upon the oxidation of membrane phospholipids, and thus reduce also its metabolites (leukotrienes and prostaglandins) that considered as important inflammatory mediators.

In broad terms, inflammation is a complex immunological defense mechanism rising either against harmful stimuli or as a result of an excessive response to a normal stimulus perceived as harmful [64]. Either way, oxidative stress plays an important role in this context as it can lead to inflammation and contribute to worsening of the condition [65]. Because of their strong antioxidant and therefore immunomodulatory activity, phenolic compounds have been proposed as compounds to use for anti-inflammatory therapy [66]. Therefore, as oxidation plays an important role in the inflammation status, anti-inflammatory products like honey, rich with antioxidant substances, have been widely used to improve inflammation and associated disorders [67]. Figure 8 briefly illustrates potential anti-inflammatory mechanisms of action for SBH based on its antioxidant activity.

This antioxidant activity would also inhibit essential enzymes responsible to the endogenous production of reactive oxygen species (ROS) during the metabolism of arachidonic acid, such as nitric oxide synthases (NOSs), cytochrome P450 (CP450), NADPH oxidase (NOX), xanthine oxidase (XO) lipoxygenase (LO), cyclooxygenases (COXs) and myeloperoxidase (MP) [68,69]. The expression of these enzymes is stimulated in the inflammatory conditions, and therefore, inhibition of these enzymes will stop ROS production that leads to reduce oxidative stress and then an anti-inflammatory effect [68,69]. Since mediators of inflammation play crucial roles in various diseases (e.g., cancer and obesity) [70,71], SBH could also rely on these mechanisms of action in modulating the inflammatory-associated diseases.

Borsato et al. reported that SBH extract has anti-inflammatory activity to reduce ear edema, probably due to a synergistic effect of its phenolic compounds (such as kaempferol and caffeic acid) [44]. It also provided a reduction in the myeloperoxidase activity along with lower leukocyte infiltration and reduced ROS production [44]. Another study on rats with selenite-induced cataract showed that there was a delay in opacification on the eye receiving treatment with SBH directly applied as a drop. [49]. Further investigation on SBH showed that the compound has even more systemic effects when administered orally to lipopolysaccharide (LPS)-induced chronic subclinical system inflammation (CSSI) rats [32]. In this context treatment with SBH significantly modulated serum inflammatory markers, such as tumor necrosis factor-alpha (TNF)-α, interleukin (IL)-1β, IL-6, IL-8, IL-10 and monocyte chemoattractant protein-1 (MCP-1), while it enhanced antioxidants like malondialdehyde (MDA) and 8-hydroxy-2′-deoxyguanosine (8-OHdG) [25,32]. However, Syam et al. observed that SBH induces mRNA expression of IL-6 in mice Balb/c strain infected with *Salmonella typhi* [42]. Altogether, these studies provided valuable insights into the potent anti-inflammatory activity of SBH, in part via its modulatory effects on reducing leukocytes infiltration and cytokines section.

### 3.3. Anti-Obesity Activity of SBH

Obesity and overweight is a worldwide health concern associated with poorer quality of life, and it is one of the leading causes of many serious diseases, including high blood pressure, diabetes and cardiovascular disease [72]. It has been reported that the high oxidative stress associated with obesity is a leading cause of inflammation [71]. Products containing high antioxidant substances showed significant anti-obesity activity [73].

In a study conducted on obese rats treated with SBH, a significant reduction was observed in body mass index (BMI), percentage of body weight gain, adiposity index, relative organ weight (ROW), liver enzymes, triglycerides and low-density lipoprotein (LDL), while the level of high-density lipoprotein (HDL) was significantly increased [34]. SBH treatment showed a reduced number of adipocyte cells, while the hepatocytes found in the liver were less prone to rupturing when treated with SBH, suggesting that SBH improved the indicators associated with obesity reduction and reduced the health risks related to obesity [34]. Another study on diabetic rats showed that SBH kept fasting blood glucose, total cholesterols, triglyceride and LDL cholesterol at low levels, whereas serum insulin and HDL cholesterol levels were increased [38]. In addition, some inflammatory markers, such as nuclear factor kappa-light-chain-enhancer of activated B cells (NF-κB), inhibitor of nuclear factor kappa-B kinase subunit beta (IKK-β), tumor necrosis factor-alpha (TNF-α) and interleukin-1 beta (IL-1β) and apoptosis marker caspase-9 showed a steady decrease following treatment with SBH, while anti-oxidative enzyme catalase was increased [38]. Together, these studies have shown that not only the antidiabetic properties of SBH as a protection agent against pancreas dysfunction, it also highlighted the potential anti-obesity activity of SBH, by maintaining the lipid profile in homeostasis and could even affect the morphological structures in the liver [34,38].

### 3.4. Anticancer Activity of SBH

It is well-known that inflammatory cells control the cancer microenvironment, while inflammation plays a crucial role in cancer progression [74,75]. It is also reported in a mechanistic review that the mechanisms for anticancer activity of honey are antioxidant, anti-inflammatory, apoptotic, immunomodulatory, TNF inhibiting, antiproliferative and provide estrogenic effects [76]. However, this study was not specific to SBH, and although there are some studies that have reported the anticancer activity of SBH, though the mechanisms of its activity are still not completely clear.

In 2016, Saiful Yazan et al. used SBH to treat rats with colorectal cancer and showed that SBH has chemo-preventative properties; this finding demonstrated by the total number of aberrant crypts and aberrant crypt foci, and crypt multiplicity all were significantly decreased [41]. Besides, their full blood count (FBC), kidney function tests (KFT) and liver function tests (LFT) were normal, indicating a non-toxic activity of SBH [41]. Another study evaluated the stability of SBH added in culture media and its proliferative effect on dental pulp stem cells (DPSCs). The results showed that SBH added to the culture media for three days resulted in a dose-dependent proliferative effect on DPSCs [35]. Another recent study by Ahmad et al. has evaluated anticancer effects of SBH on malignant glioma (U-87) cells [33]. By using cell proliferation and cells death assays, the authors demonstrated that SBH’s cytotoxic effect on this glioma cell line was time and dose-dependent. SBH also induced nuclear shrinkage, chromatin condensation and nucleus fragmentation, indicating that cellular changes were consistent with the apoptotic characteristics of the cells [33]. Taken together, these studies show exciting initial observations indicating that SBH may serve as a potential therapy for cancers and prompts the need for further investigation.

### 3.5. Antimicrobial Activity of SBH

Another potential exciting use of SBH could be in the context of antimicrobial activity. It was reported that SBH has a broad-spectrum of antimicrobial effects against four types of Gram-positive bacteria: *Bacillus subtilis, Micrococcus luteus, Bacillus megaterium* and *Bacillus brevis*, as well as two Gram-negative bacteria: *Escherichia coli* and *Pseudomonas syringae* [47]. These observations were further corroborated by Massaro et al., who studied the antibactericidal effects of a *Tetragonula carbonaria* honey on *Staphylococcus aureus* and *Klebsiella pneumoniae*. These bactericidal effects were attributed to the high concentrations of hydrogen peroxide and phenolic extracts in SBH as measured by gas chromatography-mass spectrometry [43]. Although the number of studies on *Apis mellifera* honey was significantly higher than those on SBH, several SBH types have been tested and presented higher antioxidant and biological activities, and relatively higher antimicrobial activity against fungus (*Candida albicans*), Gram-negative (*E. coli, K. pneumoniae* and *Salmonella typhimurium*) and Gram-positive (*S. aureus, Listeria monocytogenes* and *Bacillus cereus*) bacteria, respectively [30].

**Figure 9:**
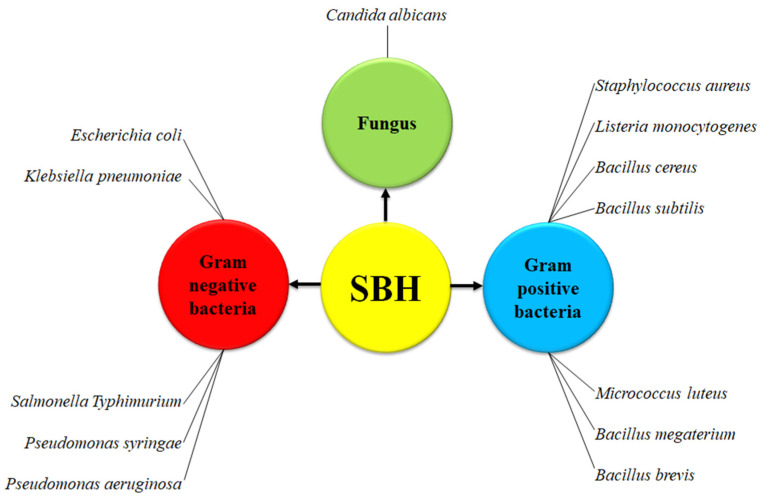
The antimicrobial activities of SBH that have been examined to date.

Another characteristic of SBH resides in the lower invertase (breaks down sucrose into fructose and glucose) and sucrose levels than *A. mellifera* honey, which has been suggested to have a strong inhibitory effect on four Gram-positive bacteria: *B. subtilis, M. luteus, B. megaterium* and *B. brevis*, and two Gram-negative bacteria: *E. coli* and *P. syringae* [48]. These findings were supported by another study that compared the antimicrobial inhibitory effects of Ethiopian SBH, *A. mellifera* honey and the most common antibiotics (vancomycin, tetracycline, kanamycin, ampicillin and methicillin) against *E. coli, S. aureus* and *K. pneumonia* [9]. Interestingly, this study has shown that the inhibitory effects of SBH against all these bacterial strains were stronger than the effects of *A. mellifera* honey and the antibiotics tested. Altogether, these interesting results suggest that SBH can be a promising candidate towards novel treatments for inflammatory conditions dependent on microbial growth. The microorganisms that have, so far, been examined following treatment with SBH are summarized in Figure 9.

Potent novel antimicrobials are also of interest to treat biofilms. The antibiofilm properties of a total of 57 SBHs were examined along with Medihoney^®^ (an antibacterial wound gel) as a reference and showed an inhibitory effect on biofilm formation in one species of honey, *Tetragonisca angustula* [40]. In addition, the identified honeys were able to destroy a *S. aureus* biofilm, mediated by two proteins, *T. angustula* biofilm destruction factor-1 (TABDF-1) and TABDF-2 [40]. These results indicate that certain honey species may be suited to develop for anti-biofilm applications and even novel wound dressings for specific (i.e., *S. aureus*) infections. In another study using *S. aureus* infected wounds, SBH (*Trigona*) was shown to synergize with the antibiotic ampicillin to inhibit *S. aureus* [37]. There was also higher bactericidal activity in the combined treatment, compared to either agent alone, including antibiotic-resistant strains [37].

### 3.6. Other Promising Properties of SBH

In addition to the conventional common properties of SBH that were mentioned above, a recent study showed that one of the components of SBH (i.e., phenylalanine) may be able to trigger the upregulation of brain-derived neurotrophic factor (*BDNF*) and inositol 1,4,5-triphosphate receptor type 1 (*ITPR1*) [26], which are genes involved in synaptic function [77,78]. Therefore, SBH displayed capabilities in improving spatial working memory, spatial reference memory and memory consolidations. Another study also suggested that SBH improves memory and reduces anxiety, in addition to its potential to reduce triglyceride, LDL, and normalize blood glucose in rats with metabolic syndrome [24].

As SBH is a good source of natural antioxidants, it was investigated as a potential anti-ageing skin treatment in human dermal fibroblast cells [27]. In this study, treatment with SBH upregulated collagen expression in both ageing and senescent (aged) fibroblast cells, and downregulated matrix metalloproteinase associated with degrading collagen, suggesting SBH may be a promising anti-ageing skin treatment may [27]. Interestingly, all of these recently discovered properties of SBH are from the Malaysian SBH (*Kelulut*) and during the last year.

### 3.7. SBH Production in Malaysia

In 2000, Malaysia produced only 5 percent of the countries honey needs, which is considered a trivial estimate compared with Malaysia’s abundant nectar resources [79]. In 2010, by providing sufficient aid, Malaysia managed to increase farm production to 284% of the countries honey needs [79]. Moreover, an immediate shift from popular Tualang (*Apis dorsata*) honey to SBH observed to be associated with successful domestication of stingless bees and an invention of a commercial MUSTAFA-Hive [80]. The hive is very well-structured; it has become easier to directly collect fresh, hygienic SBH from an intensive farm with systematic locking that protects the hives [80]. We estimated that using the MUSTAFA-hive in Malaysian stingless bee farms improves the implementation of standard operating procedure (SOP) in commercial meliponiculture, and also promote exploring the benefits of SBH in medical research. At present, 15 out of 26 studies on the medicinal properties of SBH around the world were conducted in Malaysia over the past 4 years (Table 1). However, there is still a continuous increase in honey imports, mainly from Australia, New Zealand, China and Iran [81]. Meanwhile, a significant and continuous decrease in honey exports was also reported in 2010–2017 [79]. Although Malaysian stingless bee honey is cost-effective, it is facing export restrictions due to its moisture content which is higher than the amount allowed according to the CODEX Standard 12-1981 (not more than 20 percent) [82]. Therefore, it is important to utilize the unique physiochemical characteristics of Malaysian SBH to increase demand and market value of the honey via different methods. This could also help the beekeepers to increase and sustain their income, and promote SBH as a promising future Malaysian commodity.

## 4. Stingless Bee Propolis (SBP)

Propolis (a resin-like substance, colloquially known as bee glue) is a substance that bees produce by mixing salivary secretions, beeswax, pollen and other resins harvested from botanical sources, mainly trees [83]. Additionally, geopropolis is a type of less malleable propolis only produced by some stingless bee species (for example, *Melipona fasciculata* and *Melipona quadrifasciata anthidioides*) and it is mixed with additional material (soil or clay) [76]. The benefits of SBP have been known for centuries and well-studied, similarly to SBH, and has shown four main medicinal properties; antioxidant, anti-inflammatory, anticancer and antimicrobial activities (Table 2). In addition, SBP has shown promising regenerative capacity, also making it a potential novel therapy for wound healing [74,83]. Although SBP is not as common as *A. mellifera* propolis, studies have also shown that SBP has higher antimicrobial activities [84]. The potential mechanisms of action of SBP and other stingless bee products are similar to those proposed in the previous sections for SBH.

### 4.1. Antioxidant Activity of SBP

**Figure 10:**
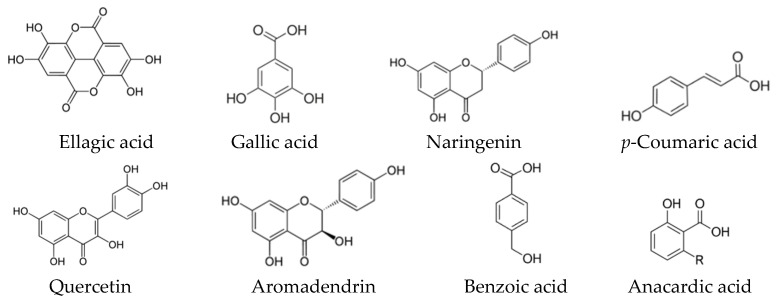
The major phenolic compounds that have been reported in SBPs [17,51,96,103].

Similar to SBH, high levels of flavonoids, phenolic acids and terpenoids have been identified in the SBP extracts, which showed promising antioxidant and antimicrobial activity [86,96]. A total of 51 phenolic compounds have been reported in SBP from *Melipona subnitida* [119]. *M. fasciculate* geopropolis was found to contain 11 compounds, mostly phenolic acids and hydrolyzable tannins, responsible for the antioxidant activity [103]. The major phenolic compounds that have been reported in different types of SBP are summarized in Figure 10.

Ethanol extracts of SBP showed antioxidant activity as they were able to scavenge free radicals in vitro and also protected erythrocytes by inhibiting oxidative hemolysis [51]. Despite some controversial reports regarding the toxic potential of SBP, Levinas et al. have performed in silico toxicity analysis involved a total of 35 SBP phenolic compounds [17]. The analysis showed that these compounds revealed low toxicity, which means that SBP is safe and not considered as a toxic product, whereas more pre-clinical studies are still required to confirm these findings.

### 4.2. Anticancer Activity of SBP

Cytotoxic activity, specifically necrosis, has been induced by SBP extracts in leukemic cell lines [51]. Apoptotic cytotoxicity has also been observed in different cancer cell lines (breast, colon, epithelial colorectal and melanoma) when treated with SBP extracts [108]. Furthermore, a dichloromethane extract of SBP displayed cytotoxic effects on head and neck squamous cell carcinoma (HNSCC) cell lines, resulting in reduced viability of these cells [94]. Novel compounds, 27-hydroxyisomangiferolic acid and 23-hydroxyisomangiferolic acid B, in the SBP extract displayed the highest levels of cytotoxicity against pancreatic cancer cells [95]. Further cytotoxic effects of SBP against canine osteosarcoma cells have been reported, with modulated cell morphologies and reduced viability of the cancer cells observed [109]. Another study found out that the piperidinic alkaloids together with C-glycopyranoside flavonoids in SBP extract were associated with apoptosis in melanoma cells, as well as inhibition of migration and invasion of these cells [90]. Further, SBP extracts demonstrated antiproliferative activity against cancer cell lines (colon, breast, hepatic and stomach), but did not affect normal cell lines (liver and fibroblasts) [107,113]. In studies mimicking the angiogenic micro-environment related to tumor development, SBP extracts demonstrated a cytotoxic effect and anti-angiogenic activity on the vascular cells [89].

Moreover, cardol (5-pentadecyl resorcinol) was the major compound identified in a fraction of SBP, exhibiting cytotoxicity against human colon, liver, gastric, lung, and breast cancer cell lines [102]. Thus, SBP has potent in-vitro anticancer activity, shown by the induction of apoptosis, and cell cycle arrest in these cell lines [102]. Kustiawan et al. also found that cardol isolated from SBP has been used to treat human colorectal adenocarcinoma cell lines, induced changes in cell morphology, increased the expression of apoptotic proteins (caspase-3 and caspase-9) and also cleavage of pro-caspase-3, pro-caspase-9 and poly (ADP-ribose) polymerase (PARP) [92]. Therefore, cardol isolated from SBP can induce cancer-induced cell death and could be a potential candidate for developing as an additional cancer therapy.

### 4.3. Anti-Inflammatory Activity of SBP

Due to their anti-inflammatory properties, SBP extracts have been used in the treatment of a murine asthma model [106]. In this model, rats treated with SBP had reduced progression of allergic inflammation as shown by decreased total cell counts in the bronchoalveolar fluid, decreased peribronchovascular inflammation and inhibition of polymorphonuclear cells into the alveolar spaces [106]. This study showed comparable anti-inflammatory effects of SBP to the positive control treatment, dexamethasone [106]. Additionally, SBP has been reported to have antinociceptive activity (inhibition of pain) due to decreased IL-1β and TNF-α [111]. Other studies have shown similar cytokine-mediated anti-inflammatory effects, as SBP extracts decreased IL-6 expression on inflamed dental pulp tissues [93].

Interestingly, different potential properties of SBP have been examined together in studies conducted by Campos et al. [99,105]. They showed that SBP has antimicrobial activity against Gram-positive and Gram-negative bacteria and fungus, as well as antioxidant activity by suppressing oxidation-induced erythrocytes hemolysis and lipid degradation [99,105]. They also reported anti-inflammatory activity by inhibition of the hyaluronidase enzyme, and necrotic cytotoxic activity against leukemia cell lines [99,105]. Another comparable study by Umthong et al. also showed the antimicrobial activities of SBP, against Gram-positive and Gram-negative bacteria and fungus, as well as antiproliferative cytotoxic activities resulting in an increase in cell death by necrosis in colon cancer cells [115].

### 4.4. Antimicrobial Activity of SBP

SBP has known antimicrobial activity against numerous microorganisms. SBP extracts have displayed potent broad-spectrum antimicrobial activity against Gram-positive bacterial (*S. aureus, Enterococcus faecalis, M. luteus, Streptococcus mutans* and *B. subtilis*), Gram-negative bacterial (*E. coli* and *Pseudomonas aeruginosa*), and yeast (*Cryptococcus neoformans* and *C. albicans*), either significantly stronger or equivalent to standard antibiotics [88,96,98,104,112,114,116].

It was reported that SBP is rich with cycloartane compounds in high enough levels to produce high antioxidant activity, which resulted in inhibitory activity against yeast α-glucosidase [87]. In addition to the antioxidant properties shown by inhibition of inflammatory enzymes [87], SBP also decreased the mutagenesis in yeast cells (*Saccharomyces cerevisiae*) [96]. In the same study, SBP extracts were more effective against gram-positive bacteria than gram-negative bacteria [96]. Furthermore, extracts of SBP demonstrated antibiofilm and antibacterial activities, by inhibiting reduced *S. mutans* growth and adherence [97]. Another study found that one of the extracted components from SBP is α-mangostin [97]. The α-mangostin (likely collected from Mangosteen) exhibited a strong antibacterial effect against both gram-positive and gram-negative bacteria, with *Staphylococcus epidermidis* being the most sensitive [100]. Similarly, the high concentrations of diterpenic acids in one extract of SBP were associated with cytotoxic activity against *Staphylococcal aureus*, but not against *E. coli* or *C. albicans* [93,106,109]. Another study has shown that cinnamoyloxy-mammeisin isolated from SBP has antimicrobial, anti-adherence and anti-biofilm activity against *S. aureus* [85].

Although SBP is not as commonly known for its antimicrobial properties as propolis from honey bees, Pino et al. have shown that the SBP from *Melipona beecheii* have higher content of trans-verbenol, α-copaene, β-caryophyllene, β-bourbonene, α-pinene, spathulenol, β-pinene and caryophyllene oxide compared to propolis from *A. mellifera*, despite both being collected from one region (Yucatán, Mexico) with similar natural flora [120]. These chemical compounds are known as potential antimicrobial agents [121,122].

## 5. Stingless Bee Cerumen

Cerumen is a mixture of propolis with the wax secreted by stingless bees before they use it for nest construction [123]. Only a few studies have been conducted, in the last decade, on stingless bee cerumen and pollen (Table 3). It has been reported that stingless bee cerumen extracts have anti-inflammatory potential as they are capable of inhibiting enzymes responsible for catalyzing the activity of pro-inflammatory mediators [124]. In this study, cerumen extracts were comparable to that of the positive control, Trolox (an antioxidant like vitamin E), though less inhibitory than honeybee propolis [124].

Stingless bee cerumen extracts have also been used as a potential anticancer agent with human breast, lung, liver, stomach and colon cancer cell lines, inducing high cytotoxicity and an apoptotic like cell morphology [15]. This study also showed that α-mangostin isolated from cerumen induced in vitro cytotoxicity against the above cell lines and in-vivo cytotoxicity against zebrafish embryos [15].

## 6. Stingless Bee Pollen

**Figure 11:**
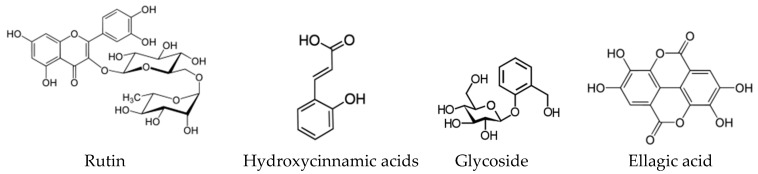
The major phenolic compounds that have been reported in stingless bee pollen [126,127,130].

Barbara et al. reported desirable nutritional properties for bee pollen extracts collected from stingless bees [130]. These extracts were reported to contain polyphenols, flavonoids and fatty acids, suggesting the antioxidant potential of stingless bee pollen [126,130]. These findings have been supported by a recent study on eight stingless bee pollen samples from different regions in the Philippines [127]. The most common phenolic compounds in stingless bee pollen are summarized in Figure 11.

There was no evidence of microbial contamination in the bee pollens examined, including *E. coli, Salmonella, S. aureus, Coliforms*, and *Clostridia*, though mesophilic microorganisms were detected [124]. Another study has shown stingless bee pollen extracts have anti-inflammatory effects by reducing mouse footpad edema and also decreasing evidence of a pain (antinociceptive) response following administration of acetic acid [130]. A recent study suggested that the anti-inflammatory effect of stingless bee pollen results from its antioxidant activity that inhibits cyclooxygenase (COX), which promotes inflammation by producing prostaglandins [126]. Furthermore, a study to detect the synergistic effect of stingless bee pollen extracts with cisplatin (a chemotherapeutic drug) on breast cancer cells showed antioxidant and antiproliferative effects of bee pollen against the cancer cells [129]. The combination of bee pollen extracts and cisplatin further reduced the proliferation of the cancer cells, compared to cisplatin alone [129]. Therefore, stingless bee pollen extracts are a potential novel cancer therapeutic agent and may be useful as an additive treatment option for chemotherapy.

## 7. Comparison of Stingless Bee Products and Their Health Benefits

Only a few studies have directly compared the potential medicinal properties of stingless bee products (Table 4). In a study by Ismail et al., each of SBH, SBP, and beebread (fermented bee pollen by lactic ferments [131]) extracts were compared for their antioxidant and anticancer effects [16]. SBP, SBH and beebread all contained phenolic content and displayed antioxidant capacity relating to the levels of phenolic content, respectively, with SBP showing the highest antioxidant capacity [16]. Additionally, treatment with SBP showed the highest growth inhibition in human breast adenocarcinoma cells, compared to SBH and beebread extracts, respectively [16]. Another study examined the cytotoxic activity of crude extracts of SBP, SBH, and bee pollen from different stingless bee species against human breast, lung, liver, gastric and colon cancer cell lines [14]. The SBP and SBH showed higher cytotoxic activities than bee pollen, with SBP exhibiting the highest overall cytotoxic activity against all cell lines, particularly from bee species *T. incisa* and *Trigona fuscobalteata* [14]. Furthermore, the potential immunomodulatory effect of SBP and SBH has been examined in a murine *S. typhi* infection model [129]. Both SBH and SBP increased FoxP3 mRNA expression (transcription factor of regulatory T cells) following infection, with the highest increase seen in the SBP treated group [132].

## 8. Bacterial Isolates from Stingless Bee Products

The bacterial species isolated from different stingless bee products have also been investigated. In a study which isolated and phenotypically characterized 41 different bacterial species from SBH, SBP and beebread, Bacillus were the predominant species found [128]. Proteolytic, lipolytic and cellulolytic extracellular enzyme activities were detected in four non-pathogenic bacterial isolates (*Bacillus amyloliquefaciens*, *Bacillus safensis*, *Bacillus stratosphericus* and *B. subtilis*) [133]. *B. amyloliquefaciens* isolates showed the highest antimicrobial activity against Gram-positive and Gram-negative bacteria [133]. Another study isolated a total of 51 symbiotic actinobacteria from stingless bees (their pollen, honey, garbage pellets and cerumen), then characterized their antibacterial effects against pathogenic human microorganisms [134]. Half of the isolates identified displayed antimicrobial activity to Gram-positive bacteria or fungi, some inhibited both, and most antibacterial isolates were from the *Streptomyces species* [132]. This study outlines the benefits to the colony, and potential human therapeutic benefits, of stingless bees acquiring actinobacteria from the areas they pollinate.

## 9. Biological Activity and Targets of Phenolic Compounds

As it is shown in the above sections, studies have discovered a variety of biological activities of stingless bee products and their compounds, while the mechanisms of action for these activities are less studied. However, the biological targets for the compounds of stingless bee products can be presumed based on previous studies on other natural products that contain similar compounds. The role of antioxidant phenolic compounds in the process of radical scavenging, to prevent damage of cellular components (diseases), has been discovered, while their antimicrobial activities still not clear. In addition to the enzymatic antioxidants that neutralize free radicals by transforming them to stable molecules (Figure 4), there are non-enzymatic antioxidants (e.g., phenolic compounds and ascorbic acid) that not only interrupt the chain reaction caused by free radicals but also can inhibit the formation of free radicals [7]. Studies on plant antimicrobial compounds showed that some of the common phenolic compounds that existed in stingless bee products, especially SBH, have strong antimicrobial potency (Table 5). These insights open the door for researchers to study the mechanisms of action for novel antimicrobial compounds in stingless bee products.

**Figure 12:**
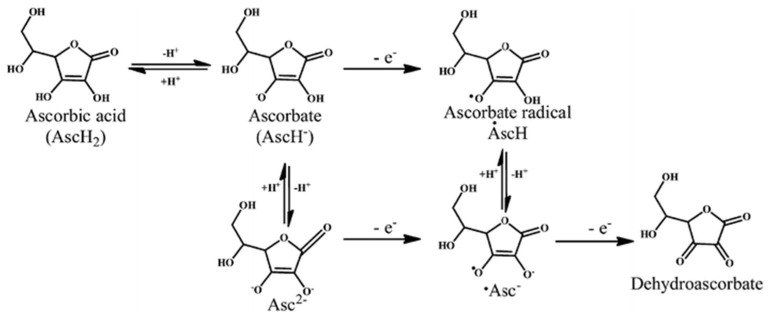
The proposed radical scavenging activity of ascorbic acid (adapted from Nimse and Pal, 2015 [143]—published by The Royal Society of Chemistry).

On the other hand, ascorbic acid (vitamin C) is a well-known antioxidant able to block the lipid peroxidation chain reaction by transforming an unpaired electron to the lipid radical (Figure 12). A study on the Afrotropical SBH produced by *Hypotrigona* sp. showed that this SBH is rich in ascorbic acid (161.69 ± 6.70 mg/kg) and has higher antioxidant properties than *A. mellifera* honey [12]. While a variety of immunomodulatory and antimicrobial properties have been suggested for ascorbic acid, studies to assess its content in stingless bee products are still required, and its antimicrobial mechanisms of action need better understanding. Furthermore, there is a gap of knowledge about the content of other potential antioxidants, such as carotenoids and tocopherol, in stingless bee products and their potential medicinal properties.

## 10. Future Directions

There is still a lack of comprehensive studies to evaluate the antioxidant potentials, quantum-chemical and molecular docking analysis of the major phenolic compounds present in each of stingless bee products. For example, a study by Fujishima et al. showed that both quercetin and gallic acid, from leaves and bark extracts of *Curatella americana* Linn, are important inhibitors of XO enzyme through its interactions with specific amino acid residues at the activity site of XO [144]. This type of studies is essential to understand the antioxidant potential of each phenolic compound with mechanisms of action. Honey is the best known primary product of stingless bees in terms of purity and availability, whereas other stingless bee products are less common, therefore here we focus on SBH and its promising opportunities.

Stingless bee species produce honey with different metabolite profiles, presenting a diverse range of biological mechanisms for the benefits of human health [145,146]. It was suggested that honey bees are the best model for gut microbiota research [147]. The gut microbiota of the honey bee provides advantages in terms of studying the systems that determine gut community composition and dynamics, as well as addressing how gut communities impact their hosts [147]. On the other hand, Razali et al. showed that the metabolite contents of the honey were varied due to different stingless bee species that might secrete different types of enzymes to the foraged nectar [146]. This was supported by Kek et al. [145], who stated that the composition of honey was probably affected by the type of bee because the honey-making process is highly dependent on the enzymes added by the bees. Since the symbiotic bacteria of insects have received increasing attention due to their prominent roles in the provision of nutrients and modulating host responses, there is a need to study the contribution of stingless bee microbial communities to the metabolites profiles and immunotherapeutic potential of their honey extracts.

Furthermore, nanoparticles show a promising role in controlling immune homeostasis during several inflammatory conditions [70,74,148]. Compared to the chemical synthesis for nanoparticles, the green synthesis (i.e., using natural products) provides advancement due to it is safer for the therapeutic intervention purposes and environment-friendly [149,150]. Although honey mediated green synthesis has been used before [151], no studies to date have utilized SBH in this process. While Kothai and Jayanthi have synthesized silver nanoparticles using SBP mediated green synthesis, and the produced nanoparticles exhibited significant anticancer activity against human lung cancer cells [101]. Therefore, it is important to study the efficiency of SBH too in the green synthesis for nanoparticles used as a treatment. Furthermore, we hypothesis that using SBH in nano-size formulations as an immune booster would be a promising approach for inflammatory-based conditions in the future. Since previous studies have shown that the effective nanoparticle size to target immune cells is approximately 50 nm [152,153], producing SBH in 50 nm size would be a novel and efficient targeting strategy for modulating the immune response in the inflammatory models including cancer microenvironment.

Additionally, information regarding the potential health benefits of SBH in medical databases is still scarce, as well as how stingless bees’ gut microbiome diversity affects the honey that they produce. In recent years, studies have focused on the importance of symbiotic bacteria due to their prominent role in nutrient acquisition and immune responses [154,155]. These bacterial communities have been best characterized in worker bees [156] and thought to consist of a core group of bacterial clades in which three major bacterial phyla (*Proteobacteria*, *Firmicutes* and *Actinobacteria*) have been found to dominate the honey bee microbiome [157,158]. Despite maintaining colony homeostasis and fitness, the contribution of their microbial communities to the metabolites profiles and immunotherapeutic properties of their extracted honey is mainly unknown. Therefore, future studies should aim to bridge this gap by focusing on the gut microbiome diversity of stingless bee species using a metagenomics approach.

Therefore, we strongly encourage future studies to perform a comprehensive metagenomics analysis on the best SBH producer species to select those capable of producing high-quality SBH with desirable properties; based on their gut microbiome. Then, it is important to do metabolomics analysis for the produced SBH, to ensure whether the expected properties exist, before proceeding towards the production of SBH in nano-size and utilize it against different inflammatory illnesses.

## 11. Conclusions

It is known that all bee products are rich with compounds that provide high nutritional and medicinal properties. However, not all bee types have been equally studied, with limited knowledge about stingless bee products compared to other honey producers. This work provides a comprehensive review of all the medicinal properties of stingless bee products that have been explored to date. To the best of our knowledge, this is the first review that addresses all these data together. The present review also provides new promising insights to improve SBH research and to achieve more utilization for its medicinal properties. In general, despite exhibiting its tremendous medicinal properties, honey has still been abandoned and disregarded in the modern pharmaceutical era and only classified under complementary medicine. Way forward, rather than treatment approach, honey should be redefined as a potential agent for disease prevention, as measured by its antioxidant properties and effectiveness in manipulating the signaling pathways in the progression of disease.

## Figures and Tables

**Table 1:** List of studies on the potential medicinal properties of stingless bee honey (SBH).

Study ID	Country of Origin	Species	Reported Properties
Arshad 2020 [24]	Malaysia	*Trigona*	Improve memory Reduces anxiety
Biluca 2020 [25]	Brazil	*Melipona marginata* *Tetragona clavipes* *Scaptotrigona bicunctata* *Melipona quadriasciata* *Tetragonisca angustula* *Trigona hypogea*	Antioxidant Anti-inflammatory
Mustafa 2019 [26]	Malaysia	*Heterotrigona itama*	Improves memory and learning
Abdul Malik 2019 [27]	Malaysia	*Heterotrigona itama*	Antiaging
Hazirah 2019 [28]	Malaysia	*Trigona*	Antioxidant
Al Kafaween 2019 [29]	Malaysia	*Trigona*	Antimicrobial
Avila 2019 [30]	Brazil	*Melipona bicolor* *Melipona quadrifasciata* *Melipona marginata* *Scaptotrigona bipuncata*	Antimicrobial Antioxidant
Selvaraju 2019 [31]	Malaysia	*Heterotrigona itama* *Geniotrigona thoracica*	Antioxidant
Ranneh 2019 [32]	Malaysia	*Trigona*	Anti-inflammatory Antioxidant
Ahmad 2019 [33]	Malaysia	*Heterotrigona itama*	Anticancer
Mohd Rafie 2018 [34]	Malaysia	*Heterotrigona itama*	Anti-obesity
Mohamad 2018 [35]	Malaysia	*Trigona itama*	Antiproliferative
Nordin 2018 [36]	Malaysia	*Trigona itama* *Trigona thorasica*	Improve wound healing
Ng 2017 [37]	Malaysia	*Trigona*	Antibacterial
Aziz 2017 [38]	Malaysia	*Geniotrigona thoracica*	Anti-obesity Antidiabetic Antioxidant
Budin 2017 [39]	Malaysia	*Trigona*	Antioxidant Improves fertility
Zamora 2017 [40]	Costa Rica	*Tetragonisca angustula* *Melipona beecheii*	Antimicrobial
Saiful Yazan 2016 [41]	Malaysia	*Trigona*	Anticancer
Syam 2016 [42]	Indonesia	*Trigona*	Anti-inflammatory
Massora 2014 [43]	Australia	*Tetragonula carbonaria*	Antimicrobial
Borsato 2014 [44]	Brazil	*Melipona marginata*	Anti-inflammatory Antioxidant
Ilechie 2012 [45]	Ghana	*Meliponula*	Antimicrobial Anti-inflammatory
Boorn 2010 [46]	Australia	*Trigona carbonaria*	Antimicrobial
Garedew 2004 [47]	Ethiopia	*Trigona*	Antimicrobial
Torres 2004 [48]	Colombia	*Tetragonisca angustula*	Antimicrobial
Patricia 2002 [49]	Venezuela	*Melipona favosa*	Anti-inflammatory

**Table 2:** List of studies on the potential medicinal properties of Stingless Bee Propolis (SBP).

Study ID	Country of Origin	Species	Reported Properties
da Cunha 2020 [85]	Brazil	*Melipona scutellaris*	Antimicrobial Anti-adherence and Anti-biofilm
Rubinho 2019 [86]	Brazil	*Melipona quadrifasciata*	Antioxidant Antimicrobial
Pujirahayu 2019 [87]	Indonesia	*Tetragonula sapiens*	Antioxidant
Abdullah 2019 [88]	Brunei Darussalam	*Heterotrigona itama*	Antioxidant Antimicrobial
Iqbal 2019 [89]	Indonesia	*Trigona*	Anticancer
Cisilotto 2018 [90]	Brazil	*Scaptotrigona bipunctata* *Melipona quadrifasciata*	Antioxidant
Brodkiewicz 2018 [91]	Argentina	*Tetragonisca fiebrigi* *Scaptotrigona jujuyensis*	Antioxidant Antimicrobial Anti-inflammatory Anticancer
Kustiawan 2017 [92]	Thailand	*Trigona incisa*	Anticancer
Sabir 2017 [93]	Indonesia	*Trigona*	Anti-inflammatory
Bonamigo 2017 [51]	Brazil	*Scaptotrigona depilis* *Melipona quadrifasciata anthidioides*	Antioxidant Anticancer
Utispan 2017 [94]	Thailand	*Trigona sirindhornae*	Anticancer
Nguyen 2017 [95]	Vietnam	*Trigona minor*	Anticancer
Santos 2017 [96]	Brazil	*Melipona orbignyi*	Antioxidant Antimicrobial
Utispan 2017 [97]	Thailand	*Trigona sirindhornae*	Antibacterial
Massaro 2015 [98]	Australia	*Tetragonula carbonaria*	Antibacterial
Campos 2015 [99]	Brazil	*Tetragonisca fiebrigi*	Antioxidant Anti-inflammatory Antimicrobial Anticancer
Sanpa 2015 [100]	Thailand	*Tetragonula laeviceps* *Tetrigona melanoleuca*	Antibacterial
Kothai 2015 [101]	India	*Tetragonula iridipennis*	Anticancer
Kustiawan 2015 [102]	Thailand	*Trigona incisa*	Anticancer
Dutra 2014 [103]	Brazil	*Melipona fasciculata*	Antioxidant
Massaro 2014 [104]	Australia	*Tetragonula carbonaria*	Antibacterial
Campos 2014 [105]	Brazil	*Melipona orbignyi*	Antioxidant Anticancer
de Farias 2014 [106]	Brazil	*Scaptotrigona postica*	Anti-inflammatory
da Cunha 2013 [107]	Brazil	*Melipona scutellaris*	Antibacterial Anticancer
Choudhari 2013 [108]	India	*Trigona*	Anticancer Antioxidant
Cinegaglia 2013 [109]	Brazil	*Melipona fasciculata*	Anticancer
Massaro 2013 [110]	Australia	*Tetragonula carbonaria*	Management of cardiovascular disorders
Franchin 2012 [111]	Brazil	*Melipona scutellaris*	Anti-inflammatory
Choudhari 2012 [112]	India	*Trigona*	Antimicrobial
Umthong 2011 [113]	Thailand	*Trigona laeviceps*	Anticancer
Liberio 2011 [114]	Brazil	*Melipona fasciculata*	Antimicrobial
Umthong 2009 [115]	Thailand	*Trigona laeviceps*	Antimicrobial Anticancer
Farnesi 2009 [116]	Brazil	*Melipona quadrifasciata* *Scaptotrigona*	Antimicrobial
Manrique 2008 [117]	Brazil and Venezuela	*Melipona quadrifasciata Tetragonisca angustula Melipona compressipes* *Nannotrigona*	Antioxidant Antimicrobial
Velikova 2000 [118]	Brazil	*Meliponinae*	Antimicrobial

**Table 3:** List of studies on the potential medicinal properties of cerumen and bee pollen from stingless bees.

Study ID	Country of Origin	Species	Reported Properties
**Cerumen**			
Paludo 2019 [125]	Brazil	*Scaptotrigona depilis*	Antimicrobial
Nugitrangson 2015 [15]	Thailand	*Tetragonula laeviceps*	Anticancer
Massaro 2011 [124]	Australia	*Tetragonula carbonaria*	Anti-inflammatory
**Bee Pollen**			
Lopes 2020 [126]	Brazil	*Scaptotrigona affinis postica*	Antioxidant Anti-inflammatory
Belina-Aldemita 2020 [127]	Philippine	*Tetragonula biroi* Friese	Antioxidant
Lopes 2019 [128]	Brazil	*Melipona fasciculata*	Antioxidant Anti-inflammatory
Omar 2016 [129]	Malaysia	*Lepidotrigona terminata*	Antioxidant Anticancer
Barbara 2015 [130]	Brazil	*Melipona mandacaia*	Antimicrobial

**Table 4:** List of studies on the potential medicinal properties of different stingless bee products.

Study ID	Country of Origin	Species	Products	Reported Properties
Ngalimat 2019 [133]	Malaysia	*Heterotrigona itama*	Honey Propolis Beebread	Antimicrobial
Cambronero-Heinrichs 2019 [134]	Costa Rica	*Tetragonisca angustula*	Adult bees and different substrates of the hive (pollen and honey storage, garbage pellets and cerumen)	Antimicrobial
Ismail 2018 [16]	Malaysia	*Trigona*	Honey Propolis Beebread	Antioxidant Anticancer
Kustiawan 2014 [14]	Thailand	*Trigona incise* *Timia apicalis* *Trigona fuscobalteata* *Trigona fuscibasis*	Propolis Bee pollen Honey	Anticancer
Usman 2016 [132]	Indonesia	*Trigona*	Honey Propolis	Anti-inflammatory

**Table 5:** List of effective antimicrobial phenolic compounds derived from plants extracts.

Compound	Mechanisms of Action	Target	References
*p*-Coumaric Caffeic acid Ferulic acid	Damaging the cytoplasmic membrane by inducing ion leakages and proton influx	*Oenococcus oeni* and *Lactobacillus hilgardii*	[135]
Myricetin	Inhibition of the intrinsic efflux pump system	*Mycobacterium smegmatis*	[136]
Luteolin	Inhibition of the intrinsic efflux pump system	*Mycobacteria spp.*	[136,137]
Naringenin Eriodictyol Taxifolin	Inhibition of β-Ketoacyl–Acyl Carrier Protein Synthase III (PfKASIII)	*Enterococcus faecalis*	[138]
Quercetin	Inhibition of the intrinsic efflux pump system	*S. aureus*	[139]
	Targeting d-Alanine:d-alanine Ligase	*Helicobacter pylori* and *E. coli*	[140]
Apigenin	Targeting d-Alanine:d-alanine Ligase	*Helicobacter pylori* and *E. coli*	[140]
Kaempferol	Inhibition of the intrinsic efflux pump system	*C. albicans* and *S. aureus*	[141,142]
